# A Central-Vein-Sign-Aware Deep-Learning Pipeline for Lesion Detection and Automated CVS Assessment on Brain SWIp: An Exploratory End-to-End Feasibility Study

**DOI:** 10.3390/jimaging12100472

**Published:** 2026-09-28

**Authors:** Petar Mladenov, Frauke Kellner-Weldon, Christoph Johann Illi, Mirko Birbaumer

**Affiliations:** 1 Institute of Natural Sciences and Humanities, Lucerne School of Engineering and Architecture, Lucerne University of Applied Sciences and Arts, 6048 Horw, Switzerland; mirko.birbaumer@hslu.ch; 2 Section of Neuroradiology, Department of Radiology and Nuclear Medicine, Neurocenter, Cantonal Hospital Lucerne, University Teaching and Research Hospital, University of Lucerne, 6000 Lucerne, Switzerland; frauke.kellner-weldon@luks.ch (F.K.-W.); christoph.illi@luks.ch (C.J.I.)

**Keywords:** multiple sclerosis, central vein sign, susceptibility-weighted imaging, deep learning, object detection, anatomical tiling, small-object detection, lesion segmentation, medical image analysis, decision support

## Abstract

The central vein sign (CVS) is a supportive imaging biomarker for multiple sclerosis (MS), but its manual assessment on susceptibility-weighted imaging with phase enhancement (SWIp) is time-consuming and reader-dependent. Existing automated methods rely on multi-contrast volumetric data and prerequisite lesion masks. We study a deliberately different formulation: a structured end-to-end pipeline operating on a single axial SWIp sequence with sparse slice-wise annotations and no prerequisite volumetric mask, combining anatomical tiling, tiled lesion detection, cross-slice reconstruction of lesion identity, and two complementary branches that independently assess the same lesion crops—direct CVS classification (Pathway A) and vein-presence gating, vein segmentation, and geometric centrality (Pathway B). A cohort of 64 clinical studies annotated by four readers was merged into a consolidated reference. The main contribution is an explicit decomposition of where such a pipeline fails. On an eight-study evaluation, the pipeline recovered 78 of 111 reference lesions (70.3%); lesion-level F1 was 0.667 for Pathway A and 0.706 for Pathway B, rising to 0.785 and 0.818 when restricted to detected lesions, and a paired test found no significant difference between the pathways (p=1.00). Detection is therefore the binding constraint, and a threshold sweep shows the associated overcounting of CVS-positive lesions is not separable from it by detector confidence alone. All operating thresholds were selected on these same eight studies, which were acquired on a single scanner, so the figures are single-centre development-set estimates rather than measures of clinical performance and are likely optimistic. Larger-cohort, acquisition-diverse independent evaluation is required before clinical use.

## 1. Introduction

Multiple sclerosis (MS) is a chronic inflammatory and demyelinating disease of the central nervous system, characterized by focal white-matter lesions [1,2]. There is no single symptom, examination finding, or laboratory test that confirms MS on its own [3,4]; diagnosis instead integrates clinical history, neurological examination, cerebrospinal-fluid analysis, and imaging, with magnetic resonance imaging (MRI) being the most important non-invasive component [5]. The 2024 revisions of the McDonald criteria formalize the diagnostic role of MRI and, for the first time, incorporate the central vein sign (CVS) as a supportive imaging biomarker [6,7].

The CVS describes a small vein running through a white-matter lesion. It is visible on susceptibility-sensitive MRI sequences and is more frequent in MS lesions than in many other white-matter diseases, so it can help distinguish MS lesions from mimics from a single examination [8]. However, applying CVS assessment in practice is difficult: identifying lesions and counting how many are CVS-positive is time-consuming when many lesions are present [8,9], inter-reader agreement is imperfect even among trained readers [10], and no single lesion-to-patient aggregation rule is universally adopted [11,12,13]. These difficulties motivate reproducible, automated image analysis that can scan a full study, flag candidate lesions, estimate CVS status, and summarize the result for expert review.

This work presents an image-analysis and deep-learning system for automated CVS assessment on susceptibility-weighted imaging with phase enhancement (SWIp) rather than a clinical validation. The system covers the full path from raw DICOM to study-level indicators and is designed to operate under the practical constraints of clinical SWIp data, where lesions and veins are small, low-contrast, and only partly visible on individual slices, and where slice-wise annotation is inherently incomplete. Its design targets the components that most affect automated CVS assessment: reliable detection of small lesions, consistent reconstruction of lesion identity across slices, and interpretable lesion-level CVS decisions that a radiologist can inspect.

The contributions of this paper are methodological. (i) We describe an anatomical tiling preprocessing step that increases the effective resolution of small lesions and reduces background before detection. (ii) We describe a cross-slice lesion reconstruction step that groups two-dimensional detections into lesion-level entities so that CVS is assessed once per physical lesion. (iii) We present two complementary lesion-centred CVS pathways: a direct classifier and a transparent vein-segmentation-and-centrality pathway, kept separate so that agreement and disagreement remain visible. (iv) We report an exploratory end-to-end feasibility evaluation on eight studies and interpret detection performance in light of the imperfect inter-reader agreement of the merged reference, quantified in Section 3.2.4. Throughout, study-level outputs are treated as research indicators and decision-support outputs; the system does not diagnose MS.

## 2. Related Work

### 2.1. Small-Object Detection via Tiling

Small objects are difficult for detection models when they occupy only a small fraction of the input. In medical imaging, the relevant signal is often surrounded by much larger regions of normal tissue, so the model sees far more background than target information, and structures with a locally similar appearance produce false positives [14]. Patch- or tile-based learning is a common response: instead of processing the whole image, it is divided into tiles, a strategy widely used in whole-slide pathology where images are too large to process directly [15]. Although SWIp slices are far smaller than whole-slide images, the same principle applies at a different scale, because a lesion only a few pixels wide on a full slice becomes larger relative to a tile. Design choices such as fixed versus content-aware tiling, tile overlap, and background suppression influence the result [14,16]. The anatomical tiling and brain-mask filtering used here serve the same purpose.

### 2.2. Segmentation in Radiology and CVS Assessment

Segmentation is used when the required output is not only a class label but also the location and shape of a structure. U-Net is a common architecture for this task because it combines a contracting path for context with an expanding path for pixel-level localization [17], and it is frequently applied to MS lesion analysis, where manual delineation is time-consuming and reader-dependent [18]. A broad survey of U-Net variants and their application across medical imaging modalities, including architectural extensions and challenges such as data heterogeneity and limited generalizability, is given in [19]. For CVS assessment, some approaches classify lesion candidates or patches directly, while others reason geometrically about whether a vein passes through the lesion centre. This distinction motivates the two-pathway design used here: one pathway predicts the CVS label directly, and the other segments the visible vein and computes a geometric centrality score.

### 2.3. Automated Central Vein Sign Assessment

Automated CVS assessment predates deep-learning classification. Dworkin et al. [20] proposed the first fully automated method: from multimodal MRI, it combined automated lesion segmentation with Frangi vesselness filtering to derive a probabilistic CVS value for each lesion and an aggregated patient-level biomarker, and on 16 participants with MS and 15 without it separated the two groups well. This established that lesion-level CVS evidence can be aggregated into a subject-level score and that end-to-end automation depends strongly on the preceding lesion-segmentation stage. Maggi et al. [21] then introduced CVSnet, a compact 3D convolutional network that classifies lesion-centred MRI patches as CVS-positive or CVS-negative and approached human lesion-level performance on a multicentre dataset; however, the lesions had to be manually preselected, so the method did not itself solve lesion detection. More recently, ALPaCA [22] proposed a fully automated multi-label 3D framework that first generates lesion candidates by voxel-wise segmentation and then applies a multi-contrast 3D convolutional network to jointly predict lesions, paramagnetic rim lesions, and the CVS, reporting strong lesion-level discrimination.

These methods rely on multi-contrast, volumetric processing and, in most cases, on prerequisite volumetric lesion masks. The framework presented here studies a deliberately different and practically relevant formulation: it operates on a single susceptibility-weighted sequence with sparse slice-wise annotations and no prerequisite volumetric lesion masks; it treats lesion localization explicitly as a small-object detection problem on axial slices; it reconstructs lesion identity across adjacent slices; and it exposes two alternative lesion-level CVS reasoning pathways together with a transparent end-to-end bottleneck analysis. It is therefore complementary to the volumetric, multimodal methods above rather than a direct competitor on dataset size, three-dimensional modelling, or absolute performance.

## 3. Materials and Methods

### 3.1. Ethics and Consent

The imaging data originate from the Department of Radiology of Lucerne Cantonal Hospital (Luzerner Kantonsspital, LUKS) and were used under the approval of the Ethics Committee of Northwest and Central Switzerland (Ethikkommission Nordwest- und Zentralschweiz, EKNZ), Project-ID 2025-02200 (“Expert reader and automated evaluation of the central vein and rim lesion sign in inflammatory diseases of the central nervous system”), granted on 24 November 2025. The study is classified as a research project involving the further use of health-related personal data. Because it constitutes further use of already-collected diagnostic imaging and health-related personal data in the absence of consent, it was approved under Article 34 of the Swiss Human Research Act (HFG) and Articles 37–40 of the Human Research Ordinance (HFV); the requirement for informed consent was accordingly waived. The approved retrospective data window is 1 January 2018 to 31 October 2025. The study was conducted in accordance with the Declaration of Helsinki. Formal statements appear in the back matter.

### 3.2. Dataset and Clinical Study Integration

#### 3.2.1. Cohort

The reference cohort for this work comprises 64 clinical brain MRI studies drawn from the institutional collection described above. The cohort was annotated in a four-reader campaign: three readers annotated all 64 studies and one reader annotated 61. All four readers were radiologists, worked from a common set of annotation instructions, and were blinded to the clinical diagnosis; during annotation they had access to both the SWIp and the corresponding FLAIR series and could consult the three-dimensional view of the institutional annotation tool. Annotating each study by several readers permits reader variability to be characterized and provides a richer training target than a single-reader reference. Demographic variables such as age and sex were not available for the present technical analysis and were therefore neither used nor estimated.

#### 3.2.2. Acquisition-Data Audit and Heterogeneity

The imaging data are stored in DICOM format, the worldwide standard for medical images, which defines both a file format (pixel data with headers) and an exchange protocol, and which assigns globally unique identifiers at the study, series, and instance levels [23]. For quality control and reproducibility, the DICOM headers of the source SWIp collection at the contributing centre were flattened into a tabular index with one row per image. The audited fields included the identity identifiers (patient, study, series, and SOP instance UIDs); modality and sequence context (series/study description, sequence name, scanning sequence, sequence variant); scanner details (manufacturer, manufacturer model name, magnetic field strength); acquisition timing and contrast parameters (echo time TE, repetition time TR, flip angle); image geometry (rows and columns, pixel spacing, slice thickness, spacing between slices, image orientation and image position vectors); and image-type and post-processing hints relevant to SWIp polarity and vein visibility.

Acquisition parameters should not be assumed uniform. The audit of the contributing centre’s SWIp collection indicates clinical heterogeneity: images were acquired on more than one scanner and at more than one field strength (predominantly 3 T, with a smaller 1.5 T contribution and a limited contribution from an additional vendor). For the SWIp series, in-plane resolution was on the order of 0.39 × 0.39 mm with a slice thickness of approximately 0.8 mm and a between-slice spacing of approximately 0.4 mm, i.e., roughly 50% slice overlap, which yields the fine through-plane sampling helpful for resolving small veins. These characteristics describe the wider institutional SWIp collection from which the cohort was drawn. The 64-study cohort itself was subsequently checked against the same index and is considerably more homogeneous than that collection: all 64 studies were acquired at 3 T on Philips scanners, 58 of them on a single model (Achieva dStream) and six on two further models of the same platform (Ingenia Elition S and Ingenia Evolution). All eight validation studies used for the end-to-end evaluation were acquired on the Achieva dStream. The cohort therefore contains no 1.5 T acquisitions and no second vendor, and the heterogeneity described above characterizes the source collection rather than the data on which the pipeline was developed and evaluated; the consequences are addressed in Section 5.

#### 3.2.3. Orientation Requirement and Sagittal Exclusions

The pipeline was developed for axial SWIp slices. Its brain-mask and quadrant-based tiling logic (Section 3.4) assume the centred-oval anatomical layout of axial acquisitions. Fifteen studies whose relevant series were acquired in sagittal rather than axial orientation were therefore excluded before tiling and detection. Mixing sagittal and axial orientations would introduce incompatible anatomical representations and could inject orientation-specific image features that are unrelated to the target task, confounding both training and evaluation. The same excluded studies were removed consistently across the detection, classification, and segmentation stages.

#### 3.2.4. Annotation Standardization and Multi-Reader Merging

Because readers used slightly different annotation styles, the data were first harmonized: ellipse annotations were converted into polygon outlines, and lesion bounding boxes were tightened to the corresponding lesion outline, reducing background around lesions and improving consistency for detection and crop-based classification. Annotations from all available readers were then merged into one consolidated file per study. Because a lesion may be annotated on several adjacent slices and by several readers, separate annotations were linked into one lesion candidate when their bounding boxes overlapped (IoU ≥ 0.1) and lay within five slices of one another. Redundant boxes on the same slice were removed in favour of the more detailed annotation. Each lesion candidate then received a single CVS label by majority vote among the readers who had annotated it and given a clear yes/no; candidates without a clear majority were labelled “unsure” rather than forced to a decision. The merged annotations provide a more complete and consistent training target than any single reader while preserving reader variability. Because the readers differed in how exhaustively they annotated lesions (annotation counts varied roughly two-fold between readers), the merged reference carries some label uncertainty, most of it at the detection stage. Agreement between readers varied considerably across stages of the annotation process. Fleiss’ κ was 0.102 for lesion selection, based on whether readers annotated the same clustered lesion candidates, 0.596 for binary CVS classification among lesions annotated by all four readers with clear yes/no labels, and 0.504 for study-level MS classification using the count-and-proportion rule of Section 3.9. Agreement was therefore substantially higher for the CVS label once lesion identity was established than for lesion selection itself. The low selection agreement should, however, be interpreted in the context of the reference construction: lesion candidates were derived from the union of all reader annotations, so regions annotated by no reader were not represented in the analysis. The selection κ therefore quantifies consistency in choosing among reader-generated lesion candidates rather than agreement across all possible lesion locations in the images. The merged reference should consequently be understood as a consensus construct rather than an exhaustive lesion inventory, and detection performance measured against it carries corresponding reference uncertainty.

#### 3.2.5. Study-Level Partitioning

All partitioning was performed at the study level: complete studies were assigned to a single partition so that slices from the same study never appear in more than one partition. Slice-level (image-level) splitting is a leakage risk in this setting, because adjacent slices of the same lesion are highly correlated; assigning whole studies to one partition removes that risk. Because no patient contributed more than one study, this study-level split also guarantees patient-level separation between partitions. The tiling, clustering, classification, and segmentation stages all inherit the same split, so crops or tiles from a given study cannot leak between training and validation.

After excluding the 15 sagittal studies (Section 3.2.3), 49 axial studies remained and were divided into 41 training studies and 8 validation studies; no separate test partition was formed. For lesion-level CVS learning, the training partition yielded 1499 labelled lesion crops carrying a binary CVS label (948 CVS-negative and 551 CVS-positive, with unsure lesions excluded), which were oversampled to balance the two classes; the validation partition was consolidated into 111 reference lesions (56 CVS-positive, 41 CVS-negative, and 14 unsure, of which 97 carry a binary label). Detector, classifier, threshold, and preprocessing choices were all made on the training and validation partitions, and the end-to-end evaluation was carried out on the same eight validation studies (Section 4.4). The eight-study evaluation is therefore a development evaluation on the validation partition, not an independent test; its numbers should be read as an internal feasibility check that is likely optimistic relative to unseen data, and we treat them accordingly throughout.

#### 3.2.6. Validation-Split Consistency Correction

During inspection of the validation split, lesions were found annotated on a single slice even though the same lesion remained clearly visible on directly adjacent slices. Such gaps penalize a correct detection on a neighbouring slice as a false positive. As a conservative, non-diagnostic consistency correction, the validation split was checked slice by slice, and reader-confirmed lesions were propagated only to adjacent slices where the same lesion was visibly still present. The correction operated at the slice-annotation (bounding-box) level and did not create any new lesion: across the eight validation studies it increased the number of reference bounding boxes from 229 to 680 (an increase of 451 boxes, per-study deltas ranging from 16 to 187), corresponding to roughly six boxes per retained lesion. This near-threefold increase in boxes reflects that readers typically annotated a lesion on a single slice even though, given the fine through-plane sampling of the SWIp series, a lesion is visible across several adjacent slices; propagation simply records the same lesion on the slices where it is present, so that a correct detection on a neighbouring slice is no longer penalized as a false positive. The propagation was carried out by the first author, who is not a radiologist, using only the image data and the existing reader annotations, with no access to any model predictions; no lesion was newly identified. A random sample of the propagated boxes was subsequently reviewed by a board-certified neuroradiologist to verify that the propagated annotations correspond to genuinely visible lesions (Section 5). One interaction should be noted for transparency: because the lesion-level reference is formed by clustering with a minimum of two boxes per cluster (Section 3.10), propagating a previously single-slice lesion onto adjacent slices can promote it from an excluded single-box annotation to a retained cluster. The corrected split therefore defines the 111-lesion reference and the 70.3% detection rate reported in Section 4.4, and these quantities are stated relative to this corrected reference. The correction was applied before the final comparative evaluation and is illustrated in Figure 1.

### 3.3. System Overview

The system is a three-stage pipeline (Figure 2). In the first stage, each SWIp slice is preprocessed and split into brain-focused tiles, a tiled detector is applied, tile predictions are mapped back to full-slice coordinates, and detections are grouped into lesion-level clusters, from which one representative crop per lesion is extracted. In the second stage, each lesion crop is assessed for CVS status by two parallel pathways. In the third stage, lesion-level predictions are aggregated into study-level indicators for radiologist review. The following subsections describe each component.

Table 1 summarizes the input, operation, and output of each stage; the following subsections describe each component in detail.

### 3.4. Slice-Wise SWIp Processing and Anatomical Tiling

A direct approach processes each full SWIp slice as a single image; slices of 560 × 560, 400 × 400, or 320 × 320 pixels are resized to 768 × 768 for the detector. A typical white-matter lesion is only about 10–25 pixels wide, so on a 560 × 560 slice it can occupy as little as 0.07% of the image. This is a small-object-detection regime in which background dominates and faint lesions must be distinguished from vessels and ventricles of similar local appearance.

Anatomical tiling addresses this. Each slice is first separated into foreground and background with Otsu thresholding [24] and then divided into smaller brain-focused tiles, each covering roughly one brain quadrant. This increases the relative lesion size in the model input and reduces irrelevant background. The dominant effect is an increase in effective resolution: a typical 182 × 218 tile resized to 768 × 768 corresponds to an approximately 3.5–4.2× zoom, whereas resizing a full 560 × 560 slice to 768 × 768 gives only about 1.4×. The detector architecture and hyperparameters are held fixed across the full-slice and tiled settings, keeping the comparison focused on the data representation rather than on a different model design.

The full-slice dataset was converted into a tiled dataset while preserving the study-level train/validation split (Section 3.2.5). A brain mask defines the brain region: Otsu thresholding separates brain from background, after which the mask is cleaned by morphological closing with a 5 × 5 kernel, retention of the largest connected component, and morphological opening with a 3 × 3 kernel; a tight bounding box is then computed and slices with a brain box smaller than 100 pixels in either dimension are skipped. The brain box is split into a 2 × 2 grid with 25% overlap in both directions, so that lesions near tile borders still appear fully in at least one tile, and tiles with less than 30% brain coverage are discarded. Each label is intersected with each tile and assigned to a tile only if at least 50% of its area falls inside, then clipped and renormalized to tile coordinates. Tiles are written as grayscale PNGs preserving bit depth, with names that encode the source slice for traceability. Full parameters are listed in Appendix A. The brain-mask construction is illustrated in Figure 3.

From 17,989 input slices, tiling produced 60,222 tiles with an average size of 182 × 218 pixels. The full validation set contained 19,083 tiles, of which 678 contained at least one lesion (the lesion-positive subset). For training, a balanced subset of 4128 tiles was formed by keeping all lesion-positive tiles and adding a capped sample of negative tiles (negatives within ±2 slices of any lesion were excluded, and the number of negatives was capped at twice the positives per study), which prevents empty tiles from dominating training. The lesion-positive validation subset contained 806 lesion instances across those 678 tiles, 26.3% more than the 638 instances counted on the 412 corresponding full slices, because lesions near a tile boundary appear in two overlapping tiles. Tiling is used only for detection; clustering, CVS classification, and study-level aggregation are unaffected. At inference, each slice is tiled with the same parameters, predictions are made per tile, and boxes are mapped back to full-slice coordinates before clustering.

### 3.5. Lesion Detection Methodology

Lesion detection was performed with modern one-stage detectors and a transformer-based detector, trained on the merged multi-reader annotations. Detection was assessed in three settings: full-slice detection, tiled detection evaluated directly on tiles, and tiled detection with predictions merged back to full-slice coordinates (with non-maximum suppression across overlapping tiles). The merge-back setting is the operative one because it reflects how the detector is used before clustering and CVS classification. Detector model selection used threshold-independent mAP; no fixed confidence operating point was imposed during selection. The detectors evaluated were YOLOv11m [25], YOLOv12m [26], YOLOv26m [27], and RT-DETR [28]. The end-to-end detection configuration is listed in Appendix D.

### 3.6. Cross-Slice Lesion Reconstruction (Clustering)

Because one physical lesion can appear on several neighbouring slices, the detector may produce several two-dimensional boxes for the same lesion. These boxes are reconstructed into lesion-level (three-dimensional) clusters before CVS assessment. Boxes on neighbouring slices are linked when they satisfy both a bounding-box overlap criterion (IoU ≥ 0.1) and a slice-gap criterion (≤5 slices), using the same spatial-linking logic as the multi-reader merge (Section 3.2.4). Clustering turns slice-level detections into single lesion entities and prevents the same lesion from receiving several potentially contradictory CVS predictions. For each cluster, the slice with the highest detector confidence is selected as the representative slice; its bounding box is expanded around the lesion centre with a padding factor of 2.0 to include surrounding vasculature, squared along the longer dimension, and resized to a fixed 320 × 320-pixel crop. This yields exactly one crop per lesion, shared by both CVS pathways. Clustering parameters are listed in Appendix D.

### 3.7. Pathway A: Direct CVS Classification

Pathway A is the main decision pathway. It classifies each 320 × 320 lesion crop directly as CVS-positive or CVS-negative with an image classifier that outputs a continuous CVS-positive probability. Training labels come from the multi-reader consensus (Section 3.2.4): each crop receives the majority-vote CVS label across the readers who detected that lesion, with unsure cases excluded, so the classifier learns from consensus judgements rather than from any single reader.

An initial three-class design added a no_lesion rejection class intended to absorb false-positive detector crops, trained with a weighted cross-entropy loss (weights 0.7 for CVS-negative, 0.85 for CVS-positive, 1.5 for no_lesion). Because this did not improve the clinically relevant CVS-positive versus CVS-negative distinction (Section 4.2), the design was simplified to two classes. The two-class model was trained on lesion crops from the detector split, with CVS-positive crops oversampled to parity and mild augmentation chosen to preserve the local vein–lesion relationship. The final model was a fine-tuned YOLOv26m classifier; full training and augmentation parameters are provided in Appendix B. The operating threshold was chosen to favour CVS-positive recall, because a missed CVS-positive lesion directly reduces the CVS-positive count used for the study-level indicator; the deployed threshold is 0.35. Implementation details are in Appendix B.

### 3.8. Pathway B: Vein Segmentation and Central-Vein Geometry

Pathway B is a more transparent alternative that decomposes the CVS decision into three stages on the same 320 × 320 representative crops, producing intermediate outputs a radiologist can inspect (Figure 4). First, a vein-presence classifier outputs the probability that the lesion crop contains a visible vein, and a probability threshold of 0.35 acts as a recall-oriented gate; crops below it do not proceed to segmentation and receive a CVS-negative Pathway B result. The operating point was selected from a validation threshold sweep. The gate is deliberately permissive because a false-negative gate decision would prevent a potentially relevant lesion from reaching the segmentation and centrality stages, whereas a false-positive gate decision primarily introduces additional downstream processing and can still be rejected by the subsequent stages. Second, a U-Net with a ResNet-34 encoder [17] produces a per-pixel vein probability map for lesions that pass the gate, which is binarized and reduced to its largest connected component. The two model targets differ in origin: the categorical has-vein and CVS labels come from the four-reader consensus, whereas the vein geometry target comes from a single selected reader annotation, because freehand polylines from several readers cannot be averaged into one reliable shape. The polyline is rasterized at fixed thickness to form the training mask, so the segmentation model is trained on an approximation of the traced vein rather than on a consensus shape—a mismatch that propagates into the centrality threshold discussed below and in Section 5. Rasterization parameters, model configuration, and binarization settings are given in Appendix C.

Third, a centrality score measures how central the predicted vein is within the crop. The crop is generated by expanding the lesion bounding box around its centre (padding factor 2.0) and squaring it, so the lesion-box centre coincides with the crop centre. The score compares $c_{\mathrm{vein}}$, the centroid of the predicted vein mask, with $c_{\mathrm{lesion}}$, taken as this crop centre, and normalizes by *r*:

(1) $$\mathrm{centrality}=1-\frac{d_{\mathrm{centroid}}}{r},\;d_{\mathrm{centroid}}=∥c_{\mathrm{vein}}-c_{\mathrm{lesion}}∥,\;r=\frac{1}{2}\sqrt{w^{2}+h^{2}},$$

where *w* and *h* are the crop dimensions, so *r* is half the crop diagonal. Because the normalization uses the crop rather than the lesion box, and $c_{\mathrm{lesion}}$ is the crop centre, the score is a crop-centred proxy for lesion centrality that is valid to the extent that the crop is centred on the lesion; higher values indicate a vein closer to the crop (and hence lesion) centre. For lesions that pass the vein-presence gate, Pathway B classifies the lesion as CVS-positive when the centrality score is at least 0.733. This threshold was selected using the Youden index on clean reader-traced vein geometry from 519 lesions.

### 3.9. Combining Pathways and Study-Level Aggregation

Both pathways answer the same lesion-level question, but with different strengths: Pathway A performs well but is opaque, while Pathway B is interpretable but limited by its segmentation stage. Rather than merging them into one score (which would hide disagreements), the pipeline keeps each pathway’s lesion-level calls, CVS-positive count, and study-level indicator separate and presents the two verdicts side by side, leaving the final interpretation to the reader. The primary study-level output is the number of CVS-positive lesions. For this study a count-based research indicator was used, combining a lesion-count criterion of the kind used in simplified CVS assessment [9,10] with a proportion criterion of the 40% type [8,11]: a study is flagged MS-indicator-positive when it has at least six CVS-positive lesions and at least 40% CVS-positive lesions. The specific combination used here was fixed a priori for this feasibility study and was not itself validated or optimized, and no single lesion-to-patient aggregation rule is universally adopted [11,13]. This threshold-and-count rule is a research indicator and decision-support output for radiologist review, not a diagnosis; the underlying operating thresholds and the choice between count-based and more sensitive percentage-based rules are deployment decisions.

One property of the rule should be noted in advance of the results. The proportion criterion becomes unstable when few lesions are detected: a study in which four clusters are reconstructed and all four are called CVS-positive yields a proportion of 100% regardless of the underlying lesion burden, and this case occurs in the evaluation reported in Section 4.4. The rule was nevertheless applied here exactly as specified above, without a minimum-detection safeguard, so that the instability remains visible in the reported results rather than masked by a post hoc correction. A deployed version should carry an explicit minimum-detected-lesion precondition—for example, applying the proportion criterion only when at least ten lesions have been reconstructed and reporting the study as indeterminate otherwise—but the appropriate minimum is itself a calibration decision that cannot be fixed on eight studies.

### 3.10. Evaluation Protocol

Detectors were evaluated with precision, recall, F1, and mAP at IoU 0.50 (mAP@0.50) and averaged over 0.50–0.95 (mAP@0.50–0.95). Classifiers were evaluated with top-1 accuracy and with threshold-dependent CVS-positive recall, precision, and CVS-negative recall. Segmentation was evaluated with Dice, IoU, precision, and recall. The vein-presence classifier was evaluated over a range of probability thresholds on 670 validation crops, with the deployed threshold selected to prioritize vein-positive recall. The centrality descriptor was evaluated separately on clean reader-traced geometry from 519 lesions using a Mann–Whitney test and rank-biserial correlation, and its decision threshold was selected using the Youden index. Two distinct evaluation units were used. Detector benchmarking (precision, recall, F1, and mAP) was performed at the bounding-box level, after tile predictions were mapped back to full-slice coordinates, matching predicted boxes to reference boxes at an IoU of 0.50. The end-to-end evaluation was instead performed at the reconstructed lesion-cluster level: the predicted detections and the reference annotations were each grouped into three-dimensional lesion clusters using the clustering rule of Section 3.6 (bounding-box IoU ≥ 0.10 and slice gap ≤ 5 on neighbouring slices; only clusters with at least two boxes retained; reference lesions under 3 mm excluded). A predicted cluster was counted as matched to a reference cluster when their boxes overlapped on at least one shared slice at an IoU of at least 0.50; at this cluster level, a matched pair with differing CVS labels counts as a classification error and an unmatched cluster as a detection error, without treating any single annotation source as absolute ground truth. The complete pipeline was then run at its final operating point on the eight validation studies (four clinically MS-positive and four clinically MS-negative), from raw SWIp DICOM through detection, clustering, crop extraction, and both CVS pathways. As noted in Section 3.2.5, these eight studies are the validation partition on which the operating thresholds and configurations were selected, so this run is a development-set feasibility evaluation, not an independent test and not clinical validation or evidence of diagnostic efficacy. To separate detection from classification, CVS performance was measured both end to end (missed lesions counted as system-negative) and in a classifier-only setting (restricted to detected lesions). Given the small evaluation set, lesion-level proportions are reported with 95% Wilson score confidence intervals for detection rate, precision, and recall. These intervals are reported to convey the uncertainty of the point estimates and to discourage over-interpretation, rather than to support formal comparisons between pathways. F1 and segmentation Dice are reported as descriptive point estimates without confidence intervals.

### 3.11. Use of Generative AI

Generative AI tools were used during the preparation of the underlying project and manuscript for code debugging and refactoring, assistance with drafting and language editing, translation, and suggestions regarding statistical analysis. Specifically, Claude Opus 4.6, 4.7, and 4.8 (Anthropic, San Francisco, CA, USA) were used for these purposes. AI-generated suggestions were treated as advisory and were reviewed, verified, and, where appropriate, modified by the authors. All methodological decisions, analyses, interpretations, and final manuscript content remain the responsibility of the authors.

## 4. Results

### 4.1. Lesion Detection

The detector was selected from four architectures on the multi-reader data in the deployed merge-back setting (Table 2). YOLOv12m gave the best overall balance: it achieved the best F1 and mAP (mAP@0.50 of 0.482) and detected more reference lesions than YOLOv11m (recall 0.643 versus 0.583) at almost the same precision. RT-DETR reached the highest recall (0.806) but at a precision of only 0.130, which is unsuitable for the automated pipeline because false detections become non-lesion crops for the CVS stage. YOLOv12m was therefore selected. The effect of anatomical tiling was architecture-dependent, and its principal benefit was on lesion recall rather than on threshold-independent mAP. For the selected architecture, tiling raised merge-back recall from 0.364 to 0.643 and mAP@0.50 from 0.298 to 0.482. However, this within-architecture mAP gain should not be read as a net improvement in best-case detection: on the mAP@0.50 metric used for model selection, the best tiled detector (YOLOv12m, 0.482) was essentially tied with the best full-slice detector (YOLOv11m, 0.485). The paired comparison (Appendix D Table A4) shows the same pattern: tiling increased recall for three of the four detectors but improved mAP@0.50 only for YOLOv12m and RT-DETR, while reducing it for YOLOv11m (0.485 full-slice versus 0.466 tiled) and YOLOv26m. The defensible summary is therefore narrower than a general endorsement of tiling: tiling shifted detector behaviour toward higher lesion recall—the property the downstream CVS stage depends on, since undetected lesions cannot be classified—and it made YOLOv12m the best-balanced detector for that stage, but it did not raise peak mAP@0.50 over the best full-slice model and its benefit was not universal across architectures. Detection nonetheless remains one of the main bottlenecks: at mAP@0.50 of 0.482 the detector finds a meaningful but incomplete share of the reference lesions, and this value must be read against the uncertainty of the merged reference, since individual readers did not annotate all lesions.

### 4.2. Pathway A Classification Results

The initial three-class setup performed only modestly (best top-1 accuracy 0.621), and the weighted loss reduced it further (Appendix B Table A1); the no_lesion class was recalled reasonably well (about 0.75) but the two clinically relevant CVS classes remained weaker, which motivated removing the rejection class. After removing no_lesion, both tested backbones improved to about 0.74 top-1 accuracy, and the final balanced YOLOv26m-cls model reached top-1 accuracy 0.753 (Appendix B Table A2).

For the deployed classifier, the operating threshold matters more than top-1 accuracy. At the default threshold of 0.50 the model is conservative, recalling CVS-negative lesions well but missing 35% of true CVS-positive lesions. The deployed threshold was therefore set to 0.35, which raises CVS-positive recall from 0.652 to 0.833 while keeping precision at 0.683 and CVS-negative recall at 0.693 (Table 3). The full threshold sweep over 596 validation crops is in Appendix B Table A3.

### 4.3. Pathway B: Vein Presence, Segmentation, and Centrality

Each stage was evaluated separately because errors accumulate along the pathway (Table 4). The vein-presence gate was evaluated on 670 validation crops, comprising 368 vein-present and 302 vein-absent cases. At the default decision point, the selected classifier reached a top-1 accuracy of 0.728, with recall of approximately 0.83 for vein-present crops and 0.60 for vein-absent crops. Because the gate determines whether a lesion proceeds to segmentation, the deployed probability threshold was lowered to 0.35 to prioritize vein-positive recall. At this operating point, vein-positive recall was 0.894, precision was 0.639, and vein-negative recall was 0.384. The resulting gate is deliberately permissive: it passes more vein-absent crops to segmentation but reduces the risk of excluding true vein-positive lesions prematurely. Vein segmentation was the weakest stage: on 373 validation crops the selected U-Net (ResNet-34 encoder) reached Dice 0.42 (IoU 0.28, precision 0.44, recall 0.44), outperforming the tested YOLO segmentation variants. The low Dice reflects the difficulty of the task: veins are very thin, so small spatial errors strongly reduce overlap; the training masks are approximations rasterized from sparse single-reader polylines at fixed thickness; and SWIp has anisotropic resolution, so the vein course is coarsely sampled across slices. The predicted masks therefore capture the approximate vein location but not a precise shape. Example 2 in Figure 4 shows a representative case in which this shape error is sufficient to change the lesion-level call. The centrality descriptor, evaluated on clean reader-traced geometry for 519 lesions, separated central from non-central veins in the expected direction (Mann–Whitney $p<10^{-5}$), although the effect was small (rank-biserial correlation 0.24). The Youden-optimal threshold was 0.733, with precision of approximately 0.74. The descriptor therefore contains a measurable signal, but the separation remains limited. Because the threshold was derived from clean reader-traced geometry and subsequently applied to vein masks predicted by the U-Net, Pathway B is treated as supporting evidence rather than as a standalone decision pathway.

### 4.4. Exploratory End-to-End Feasibility Evaluation

The complete pipeline was run on the eight validation studies. As stated in Section 3.2.5, these are the same studies on which the detector, classifier, and thresholds were selected, so this is a development-set feasibility check rather than an independent test, and the figures below are likely optimistic relative to unseen data. Reference boxes were clustered with the same lesion-level logic as the pipeline (clusters with at least two boxes; lesions under 3 mm excluded), giving 111 reference lesions (56 CVS-positive, 41 CVS-negative, 14 unsure). The pipeline detected 78 of the 111 reference lesions, corresponding to a lesion-level detection rate of 70.3% (95% Wilson CI, 61.2–78.0%), and produced 126 clusters overall, of which 46 were not matched to any reference lesion. (Eighty predicted clusters matched a reference lesion, but these covered 78 distinct reference lesions, two of which were each matched by two predicted clusters.) These 46 unmatched clusters should not automatically be interpreted as false detections. A sensitivity analysis over the detector confidence threshold is reported in Appendix D Table A6 and discussed in Section 5. Because the readers differed substantially in which lesions they annotated (Fleiss’ κ of 0.102 for lesion selection, Section 3.2.4), the merged reference does not constitute an unquestionable inventory of all lesions visible in the images. Some unmatched clusters may represent genuine lesions absent from the merged annotations, whereas others may result from clustering or matching errors or may represent ambiguous candidates. Distinguishing among these possibilities would require targeted radiological review of the unmatched clusters and was outside the scope of this study. The dominant limitation is that almost one third of the annotated lesions did not reach the CVS stage; because missed lesions cannot be classified later, detection errors directly reduce the reportable CVS-positive count.

To separate detection from classification, CVS performance on the 97 binary reference lesions was measured both end to end and classifier-only (Table 5). When missed lesions were excluded, F1 increased from 0.667 to 0.785 for Pathway A and from 0.706 to 0.818 for Pathway B. This conditional analysis indicates that missed detections were a major contributor to the lower end-to-end performance. However, the classifier-only estimates apply only to lesions that were successfully detected and therefore describe performance on a selected subset. The observed differences between the pathways are small and, as shown below, are not statistically distinguishable from chance. The two pathways behave differently: Pathway A is more specific (precision 0.838), making fewer false CVS-positive calls but missing more true positives, whereas Pathway B is more sensitive (recall 0.643 end to end, 0.857 classifier-only), finding more CVS-positive lesions but producing more false positives. The pathways produced different calls for 22 of the 78 detected lesions. Five of these fall on lesions labelled unsure in the reference and are therefore not scored; the remaining 17, all among the 66 binary-labelled detected lesions, were resolved in favour of Pathway B nine times and Pathway A eight times. A paired McNemar test on these discordant pairs gave p=1.00 (exact, two-sided), so the difference in lesion-level accuracy between the pathways is not distinguishable from chance. The same result is obtained end to end, because lesions missed by the detector are scored identically for both pathways and contribute only concordant pairs. Example 2 in Figure 4 illustrates one of the eight discordant lesions resolved in favour of Pathway A. The disagreements were nonetheless directional: among CVS-positive lesions Pathway B was correct in seven of nine discordant cases, whereas among CVS-negative lesions Pathway A was correct in six of eight, consistent with the sensitivity–specificity trade described above and with the limited specificity of the centrality descriptor. These counts should be read with the caveat that the discordant lesions are unevenly distributed across the eight studies—six arise from a single study—so the pairs are not fully independent. Although Pathway B shows a higher F1 than Pathway A in both settings, this should not be read as evidence that Pathway B is the stronger pathway: the paired comparison reported above found no significant difference, the estimate rests on a small set of detected lesions, the centrality descriptor’s effect size is small (rank-biserial 0.24), and its threshold was selected on clean reader-traced geometry rather than the predicted masks it is applied to.

At the study level, the most relevant output is the number of CVS-positive lesions per study (Table 6). Both pathways reported more CVS-positive clusters than the reference (80 for Pathway A and 85 for Pathway B versus 56), reflecting unmatched detections and recall-oriented operating choices. Pathway A used a CVS-positive classification threshold of 0.35, while Pathway B used a permissive vein-presence gate of 0.35 before applying the separate centrality criterion of 0.733. When the count-based research indicator (≥6 CVS-positive lesions and ≥40% CVS-positive) was applied, the Pathway A indicator was positive for all four clinically MS-positive studies and negative for all four clinically MS-negative studies. The Pathway B indicator matched the clinical label in seven of eight studies, with one indicator-positive pattern in an MS-negative study (Study 5). These findings demonstrate the feasibility of aggregating lesion-level predictions into an interpretable study-level output. However, they should not be interpreted as an estimate of study-level discriminative performance, because the detector and operating thresholds rule were configured using these same eight studies and the rule was not independently validated. Table 6 therefore illustrates the behaviour of the configured pipeline on the development set; validation on an independent and substantially larger cohort is required. These outputs are exploratory research indicators and not diagnostic classifications.

## 5. Discussion

This work assembles an SWIp image-analysis pipeline in which slice-wise preprocessing, anatomical tiling, tiled detection, cross-slice lesion reconstruction, and two lesion-centred CVS pathways operate end to end and produce study-level research indicators for review. The methodological findings are consistent and mutually reinforcing. Anatomical tiling improved the effective resolution of small lesions and, for the selected architecture, substantially increased detection recall over full-slice inference. Its benefit was recall-oriented and architecture-dependent rather than a uniform gain: peak mAP@0.50 was comparable between the best tiled and best full-slice detectors. Among the evaluated detectors, tiled YOLOv12m gave the best recall/precision balance for the downstream CVS stage, which is the reason it was selected. Direct CVS classification (Pathway A) was accurate and, at a recall-favouring threshold, produced a usable operating point, while the transparent pathway (Pathway B) added interpretability through an inspectable vein mask and a geometric centrality score. Keeping the two pathways separate preserved their disagreements, which is more informative for a reviewing radiologist than a single merged number.

All readers were weighted equally in the merge. Annotations were linked spatially and each lesion candidate received its CVS label by unweighted majority vote among the readers who annotated it, with ties labelled unsure. Equal weighting was chosen because all four readers were radiologists working from a common instruction set and blinded to the clinical diagnosis, so no principled basis existed for ranking them; any weighting by seniority or by annotation volume would have encoded an untested assumption about reader accuracy into the training target. The cost of this choice is that it treats the roughly two-fold difference in how exhaustively readers annotated lesions as reader variability rather than as a difference in reliability. A weighted or reliability-adjusted merge—for example weighting by agreement with the consensus, or modelling reader sensitivity explicitly—is a reasonable alternative and was not evaluated here.

Several limitations bound the interpretation, and they should be read together with the paper’s positioning as a feasibility study. First, the end-to-end evaluation used only eight studies, and those eight are the validation partition on which the detector, classifier, and operating thresholds were selected; there was no separate test set, so the evaluation is a feasibility check whose numbers are likely optimistic relative to unseen data. An independent cohort is the necessary next step and is left for future work. The study-level indicators are encouraging but constitute an early feasibility signal, not clinical validation or evidence of diagnostic efficacy.

Second, lesion detection remains the dominant performance limit: the pipeline detected 70.3% of the merged reference lesions, and because later stages only process detected lesions, missed lesions directly cap the reportable CVS-positive count.

Third, the reference annotations are themselves uncertain, because readers differed substantially in which lesions they annotated and, for some lesions, in the assigned CVS label. Detection rates and unmatched clusters must therefore be interpreted in the context of human annotation variability rather than against an absolute ground truth. The merged annotations are best understood as a consensus reference that reduces individual-reader variability but does not eliminate uncertainty, particularly regarding which lesions should be included. A specific qualification concerns the validation-split consistency correction (Section 3.2.6). The propagation was performed by a non-radiologist and increased the box-level reference from 229 to 680 boxes, and its effect is not confined to the box level: because the lesion-level reference retains only clusters with at least two boxes (Section 3.10), propagation also determines which lesions enter the 111-lesion reference at all. It therefore influences both the numerator and the denominator of the reported 70.3% detection rate. To bound this risk, a random sample of 50 propagated boxes (11% of the 451 added boxes, drawn across all eight validation studies) was reviewed by a board-certified neuroradiologist (F.K.-W.), who confirmed a visible lesion in 49 of 50 boxes (98%, 95% Wilson CI 0.895–0.996). The single remaining box was not judged incorrect but indeterminate on SWIp alone, requiring FLAIR correlation for a definitive call. Taking the lower confidence bound as a conservative case, the expected number of propagated boxes not corresponding to a visible lesion is bounded at roughly 47 of the 451 added, which would affect the box-level reference but is unlikely to alter the composition of the 111-lesion reference materially, since a lesion is retained only when at least two boxes support it. A larger verification sample would tighten this bound but, at 98% observed agreement, would not change its interpretation. The propagated reference is therefore consistent with radiological reading at the sampled level, but it remains a consistency correction rather than an independent annotation; the detection rate should be read as conditional on this corrected reference, and an independent cohort annotated multi-slice by radiologists from the outset remains necessary.

Fourth, Pathway B is limited by its segmentation stage (Dice 0.42) and by a centrality descriptor with only modest separation (rank-biserial 0.24); the centroid-based descriptor can also penalize long veins that pass through the centre but extend to one side.

Fifth, there is a train–inference gap. The classifiers were trained on clean reader-labelled crops but applied to noisier detector-generated crops. In Pathway B, the centrality threshold of 0.733 was selected from clean reader-traced vein geometry but was applied in the end-to-end pipeline to masks predicted by the U-Net. Differences between clean and predicted geometry may therefore reduce the reliability of the centrality decision in deployment. Because the centrality threshold was fitted to clean geometry, Pathway B’s reported lesion-level performance should be regarded as provisional and not as performance under a properly calibrated deployment setting. Recalibration on predicted-mask centrality scores requires an independent cohort. Recalibrating the threshold on predicted-mask centrality scores would require a partition on which the threshold had not already been fitted; performing such recalibration on the present validation studies would introduce an additional threshold selected on the evaluation set and compound the development-set circularity discussed above. We therefore leave this recalibration to evaluation on an independent cohort.

Sixth, the cohort is acquisition-homogeneous in exactly the respects that govern vein conspicuity on susceptibility-weighted imaging. Although the contributing centre’s wider SWIp collection spans more than one field strength and vendor (Section 3.2.2), all studies used here were acquired at 3 T on Philips scanners, and all eight validation studies on a single model. Whether detection or CVS classification degrades at 1.5 T, on other vendors’ SWIp implementations, or under different echo times and through-plane sampling could not be assessed and remains entirely open. Because vein conspicuity depends directly on field strength, echo time, and voxel geometry, this is a substantive constraint on external validity rather than a formality: the reported figures should be read as single-vendor, single-field-strength, and in the validation partition single-scanner estimates. An independent evaluation cohort should therefore be acquisition-diverse as well as larger.

Two features of Table 6 further qualify the apparent agreement between the indicator and the clinical labels. First, because the count criterion is a lower bound, the overcounting visible in Table 6 (80 and 85 predicted CVS-positive clusters against 56 in the reference) moves studies toward indicator-positive; the agreement observed for the four MS-positive studies is therefore not independent of the pipeline’s false-positive behaviour. Second, Study 8 shows how the proportion criterion behaves when detection is poor: only four clusters were reconstructed against 14 reference lesions (none CVS-positive), and Pathway B called all four CVS-positive, giving a proportion of 100% in a clinically MS-negative study. Only the count criterion prevented an indicator-positive call. A minimum-detected-lesion precondition on the proportion criterion is therefore a necessary component of any deployed version of this rule (Section 3.9), though its threshold cannot be calibrated on the present sample.

The extent to which this overcounting is separable by detector confidence alone can be bounded directly. The deployed pipeline applies a detection confidence threshold of 0.10; sweeping this threshold upward over the eight-study run, with every other pipeline parameter held fixed, gives the behaviour in Appendix D Table A6. A modest increase to 0.15 removes seven unmatched clusters at no cost in detection: the same 78 reference lesions are recovered, the study-level indicator is unchanged in all eight studies, and the CVS-positive totals fall from 80 to 74 for Pathway A and from 85 to 79 for Pathway B. Beyond that point the trade reverses sharply. At 0.20 the detection rate falls from 70.3% to 55.9%, and the study-level indicator that changes is a false negative rather than a correction: the Pathway B indicator turns negative for one clinically MS-positive study, while the indicator-positive pattern in the MS-negative study discussed above persists. Confidence thresholding therefore removes a genuine detection before it removes the spurious study-level call, and cannot separate overcounting from the detection recall that is already the binding constraint. This is precisely the limitation that a dedicated rejector would address, since a rejector trained on detector output could discriminate non-lesion crops from genuine low-confidence lesions rather than removing both indifferently.

A direct consequence of the unmatched-cluster analysis is that the pipeline currently has no mechanism to reject non-lesion crops before CVS assessment. The three-class design with a no_lesion rejection class was abandoned because it degraded the clinically relevant CVS-positive versus CVS-negative distinction (Section 4.2), but that classifier was trained on clean reader-labelled crops rather than on the detector-generated crops it would have to reject at inference. The consistent next step is therefore a dedicated false-positive rejector trained directly on detector output: crops produced by the deployed detector on the training partition, labelled as lesion or non-lesion, so that the rejector learns the actual false-positive distribution of the detector rather than an approximation of it. Such a stage would act between clustering and CVS assessment and would directly address the overcounting observed in Table 6, at the cost of an additional operating point to calibrate. This was not implemented or evaluated here.

Because lesion detection is the binding constraint, it is worth stating both what might raise detection recall and what that would be worth. Three routes are available without changing the annotation format. First, the tiling geometry was fixed at a 2 × 2 grid with 25% overlap and was not itself optimized; finer grids, larger overlaps, or content-aware tile placement would increase the effective magnification of small lesions further, at a proportional cost in inference time. Second, the four evaluated architectures behaved differently enough—RT-DETR reached a recall of 0.806 at a precision of 0.130, whereas YOLOv12m reached 0.643 at 0.459—that an ensemble or a cascaded high-recall proposal stage followed by the rejector described above could plausibly recover lesions that the single selected detector misses. Third, detection currently operates slice-wise and recovers lesion identity only afterwards by clustering; a detector operating directly on adjacent-slice stacks could exploit the fine through-plane sampling of the SWIp series at the detection stage rather than after it.

The benefit of such improvements can be bounded. Because undetected lesions are scored as system-negative and therefore contribute only false negatives, end-to-end lesion-level recall factorizes as the product of the detection recall on CVS-positive reference lesions and the classifier-only recall. The pipeline recovered 42 of the 56 CVS-positive reference lesions, a detection recall of 0.750 on this subset—higher than the 70.3% rate over the reference as a whole—and 0.750 × 0.738 = 0.554 reproduces the end-to-end recall reported for Pathway A in Table 5. Holding classifier performance fixed, raising detection recall on CVS-positive lesions to 0.85 would give an end-to-end recall of approximately 0.63 for Pathway A and 0.73 for Pathway B, and to 0.90 would give approximately 0.66 and 0.77; perfect detection would reproduce the classifier-only figures of 0.738 and 0.857, which are therefore the ceiling that detection improvement alone can reach. These projections are optimistic, because they assume the classifiers perform on newly recovered lesions as they do on currently detected ones, whereas the lesions the detector currently misses are the smaller and lower-contrast ones that are also harder to classify. They nonetheless indicate that improving detection alone would not eliminate the remaining classification errors, and that corresponding improvements in the CVS-classification stage would also be required.

A recurring root cause is the two-dimensional annotation format. Slice-by-slice annotation could not encode lesion identity across slices or full three-dimensional vein geometry, so lesion identity had to be reconstructed afterwards by clustering and the central vein could only be recorded as short two-dimensional polylines. Future work should therefore evaluate a volumetric annotation workflow—using offline tools that support volumes and, potentially, prompt-based propagation—in which lesions are annotated as objects with stable identity and veins as three-dimensional centrelines. This was not tested here and would require a dedicated feasibility study; anisotropic SWIp voxels also limit how reliably veins can be assessed in three dimensions. Beyond annotation, the centrality metric should be revisited with alternatives (for example, the closest vein point to the lesion centre, or a vein-centreline course), the data-preparation steps should be consolidated into one reproducible retraining workflow, and a radiologist should review a sample of unmatched clusters to estimate true detector precision. Most importantly, the system should be evaluated on a substantially larger and acquisition-diverse cohort, spanning field strengths and vendors, with the CVS threshold calibrated for the intended setting, before any use beyond research is considered; a comparison against volumetric, multi-contrast methods such as ALPaCA [22] on a shared cohort would further help position the single-sequence SWIp formulation studied here.

## 6. Conclusions

We presented a central-vein-sign-aware deep-learning pipeline for brain SWIp that runs end to end—from raw DICOM through slice-wise preprocessing, anatomical tiling, tiled lesion detection, cross-slice lesion reconstruction, and two complementary lesion-centred CVS pathways—to study-level research indicators for radiologist review. The value of assembling the full path lies less in the individual component scores than in locating where such a pipeline fails. On the evaluated components, anatomical tiling shifted detection toward higher lesion recall without raising peak mAP@0.50 over the best full-slice detector, direct CVS classification reached a usable operating point, and the transparent segmentation-and-centrality pathway provided interpretable but currently supporting evidence; a paired comparison found no significant difference between the two pathways. In an exploratory eight-study evaluation, the pipeline detected 78 of 111 reference lesions and its study-level indicators tracked the clinical labels on the same studies used to configure the pipeline, which is a consistency check rather than evidence of discriminative performance. Lesion detection is the binding constraint: it caps end-to-end sensitivity directly, and the associated overcounting of CVS-positive lesions cannot be separated from it by detector confidence alone, which points to a rejector trained on detector output as the next concrete step. These results demonstrate technical feasibility rather than clinical validity—the reference annotations carry real reader variability, the cohort is single-centre and single-field-strength, and the system does not diagnose MS. Larger-cohort, acquisition-diverse evaluation, threshold calibration, and improved annotation are the necessary next steps.

## Figures and Tables

**Figure 1:**
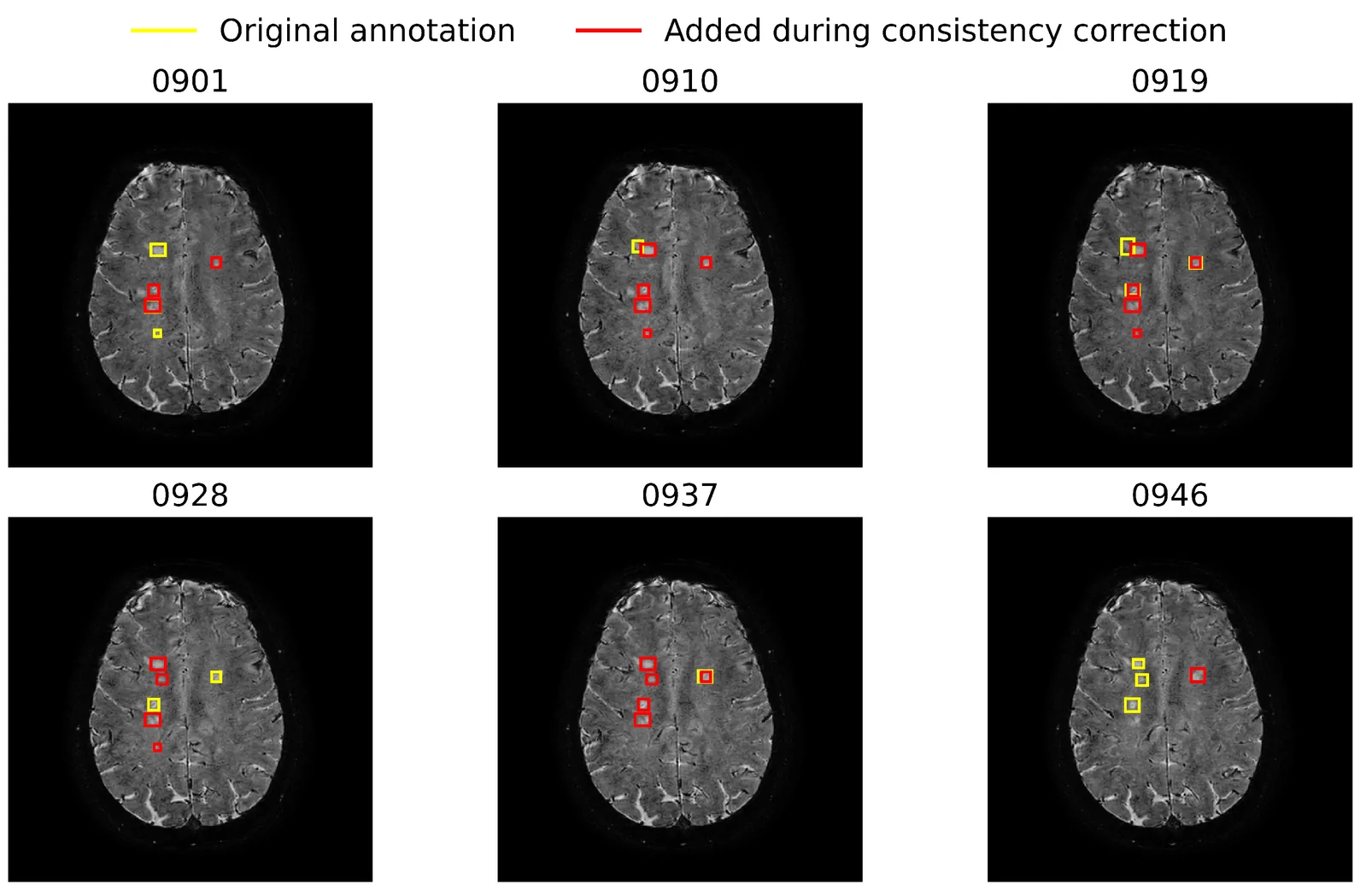
Validation-split consistency correction across adjacent SWIp slices, shown for one illustrative study. Six consecutive SWIp slices are shown; in the file naming convention used here, adjacent slices differ by 9 in their slice index (e.g., 0901, 0910, 0919). Yellow boxes denote original lesion annotations, and red boxes denote lesion annotations added during the consistency correction. No new lesions are introduced; the correction uses only image data and existing reader annotations.

**Figure 2:**
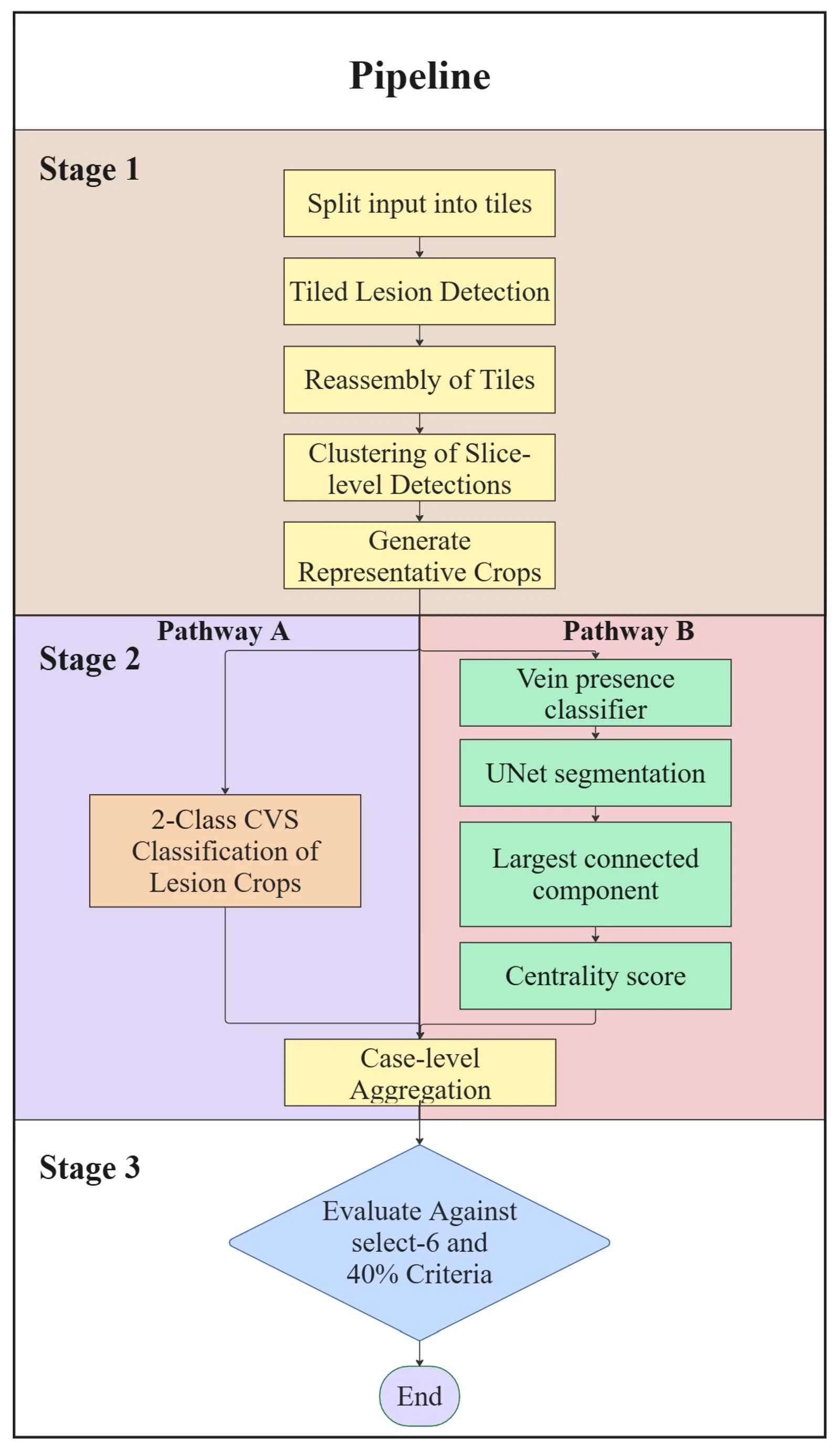
Overview of the proposed pipeline. In Stage 1, axial SWIp slices undergo preprocessing and anatomical tiling, followed by tiled lesion detection, mapping of detections back to full-slice coordinates, cross-slice lesion reconstruction, and extraction of one representative crop per reconstructed lesion. In Stage 2, each lesion crop is assessed independently by direct CVS classification (Pathway A) and by vein-presence gating, vein segmentation, and geometric centrality assessment (Pathway B). In Stage 3, lesion-level predictions are aggregated into study-level research indicators for radiologist review.

**Figure 3:**
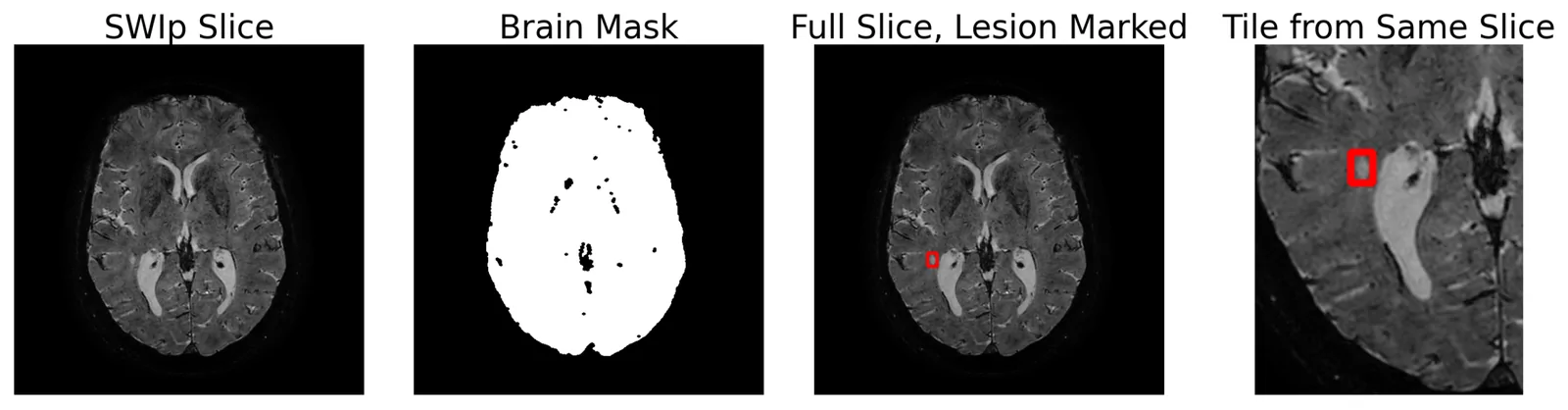
Brain-mask construction for anatomical tiling. Otsu thresholding separates brain from background; morphological closing and opening followed by largest-connected-component selection yield a clean brain mask, whose bounding box defines the region subsequently split into overlapping tiles. The red bounding box indicates an annotated lesion.

**Figure 4:**
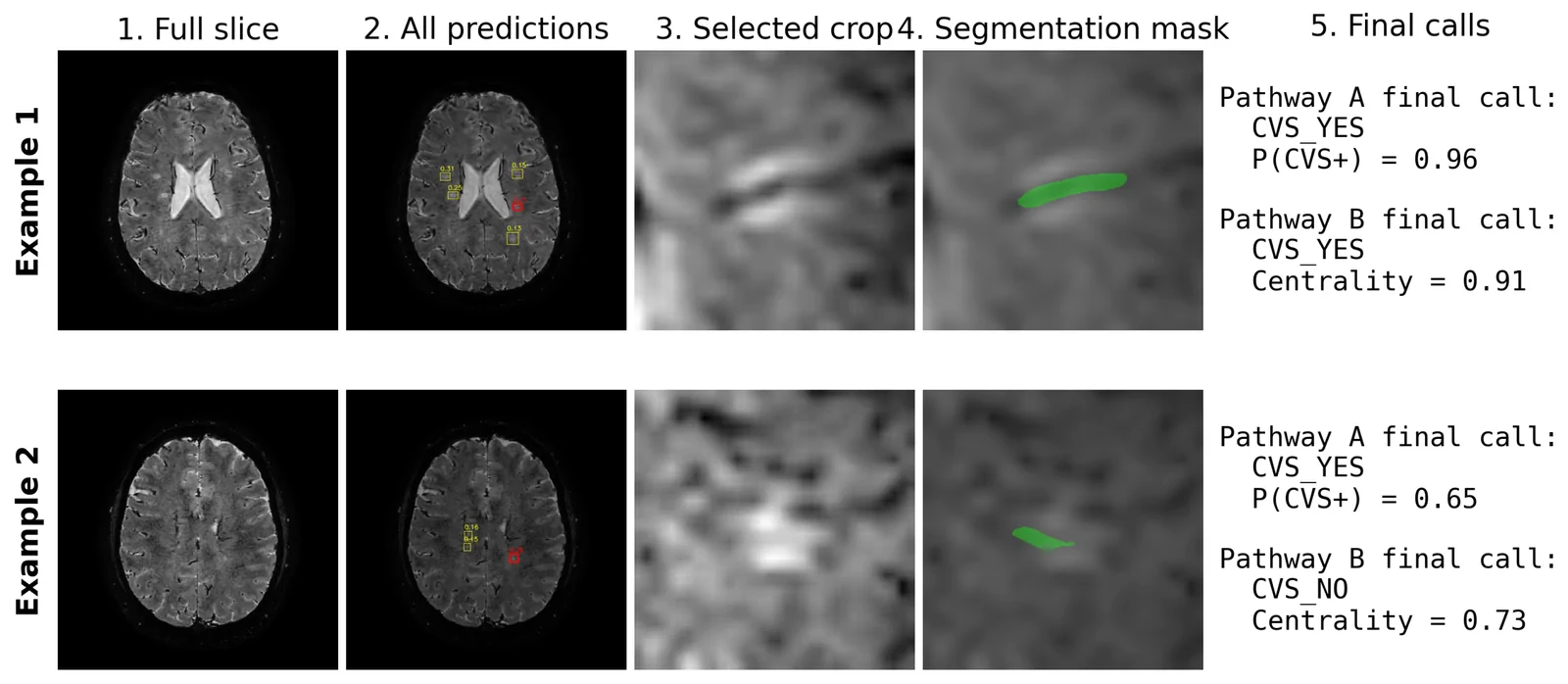
Pathway B applied to two lesions, showing the intermediate outputs a radiologist can inspect. For each lesion, (**1**) the full axial SWIp slice with all detections; (**2**) the selected cluster in red; (**3**) the extracted 320 × 320 lesion crop; (**4**) the U-Net vein segmentation (green mask represents vein segmentation); (**5**) the resulting centrality score and the final call, together with the Pathway A call for the same crop. (**Example 1**) Successful case. (**Example 2**) Failure case: a lesion the reference labels CVS-positive, which Pathway A calls correctly (P(CVS+)=0.652) but Pathway B calls CVS-negative. The vein-presence gate passes the crop (P(vein)=0.888) and segmentation localizes the vein, but recovers a compact fragment rather than its full course; the centroid of that fragment falls marginally off the crop centre, giving a centrality of 0.732 against a decision threshold of 0.733. The example illustrates the train–inference gap discussed in Section 5: the threshold was selected on clean reader-traced vein geometry, whereas the masks it is applied to have a segmentation Dice of 0.42 and capture approximate vein location rather than precise shape.

**Table 1:** Summary of the pipeline stages, showing the input and output of each component, the model or operation applied, and the section in which it is described. Thresholds are the deployed values listed in Appendix D Table A5.

Step	Input	Operation/Model	Output
Stage 1—Detection and lesion reconstruction			
Preprocessing	Axial SWIp DICOM slice	Otsu brain mask, morphological cleaning, largest connected component (Section 3.4)	Brain bounding box
Tiling	Slice + brain box	2 × 2 grid, 25% overlap; tiles below 30% brain coverage discarded (Section 3.4)	Brain-focused tiles
Detection	Tile	Tiled YOLOv12m, confidence 0.10 (Section 3.5)	Lesion boxes per tile
Merge-back	Tile boxes	Mapping to full-slice coordinates, cross-tile NMS at IoU 0.30 (Section 3.5)	Slice-level boxes
Clustering	Slice-level boxes	Linking on neighbouring slices at IoU ≥ 0.10, gap ≤ 5; minimum two boxes per cluster (Section 3.6)	Lesion-level clusters
Crop extraction	Cluster	Highest-confidence slice, box padded ×2.0, squared, resized (Section 3.6)	One 320 × 320 crop per lesion
Stage 2—CVS assessment (both pathways use the same crop)			
Pathway A	Lesion crop	YOLOv26m-cls two-class classifier, threshold 0.35 (Section 3.7)	CVS-positive/negative
Pathway B, gate	Lesion crop	Vein-presence classifier, threshold 0.35 (Section 3.8)	Proceed or CVS-negative
Pathway B, segmentation	Gated crop	U-Net (ResNet-34 encoder), mask threshold 0.50, largest connected component (Section 3.8)	Binary vein mask
Pathway B, centrality	Vein mask	Normalized vein-centroid-to-lesion-centre distance, Equation (1), threshold 0.733 (Section 3.8)	CVS-positive/negative
Stage 3—Study-level aggregation			
Aggregation	Lesion-level calls	≥6 CVS-positive lesions and ≥40% CVS-positive, applied separately per pathway (Section 3.9)	Two study-level indicators

**Table 2:** Detector comparison in the deployed merge-back setting. YOLOv12m gave the best balance for the downstream CVS stage; RT-DETR found more lesions but produced too many false detections.

Merge-Back Model	Precision	Recall	F1	mAP@0.50	mAP@0.50–0.95
YOLOv12m (selected)	0.459	0.643	0.536	0.482	0.170
YOLOv11m	0.467	0.583	0.519	0.466	0.150
YOLOv26m	0.287	0.414	0.339	0.248	0.069
RT-DETR	0.130	0.806	0.224	0.432	0.145

**Table 3:** Effect of the Pathway A operating threshold. Lowering the threshold from 0.50 to 0.35 improves CVS-positive recall from 65% to 83% while keeping precision and CVS-negative recall at usable levels.

Threshold	CVS-Positive Recall	Precision	CVS-Negative Recall
0.35 (deployed)	0.833	0.683	0.693
0.50 (default)	0.652	0.758	0.834

**Table 4:** Pathway B stage-wise results. The gate is lenient, segmentation is limited, and the centrality descriptor carries a small but significant signal.

Stage	Result
Vein-presence gate (670 crops)	Default top-1 accuracy 0.728; at deployed threshold 0.35: vein-positive recall 0.894, precision 0.639, vein-negative recall 0.384
Vein segmentation (373 crops)	U-Net/ResNet-34: Dice 0.42, IoU 0.28, precision 0.44, recall 0.44
Centrality (519 lesions)	Mann–Whitney $p<10^{-5}$; rank-biserial 0.24; Youden threshold 0.733; precision ≈ 0.74

**Table 5:** End-to-end versus classifier-only CVS performance on the 97 binary reference lesions. Precision and recall are reported with lesion-level 95% Wilson score confidence intervals; F1 and accuracy are descriptive point estimates. Classifier-only results are conditioned on successful lesion detection. The increase in classifier-only F1 indicates that missed detections were a major contributor to the lower end-to-end performance.

Setting	Pathway	Counts TP/FN/FP/TN	Precision (95% CI)	Recall (95% CI)	F1	Acc.
End to end	A	31/25/6/35	0.838 (0.689–0.923)	0.554 (0.424–0.676)	0.667	0.680
End to end	B	36/20/10/31	0.783 (0.644–0.877)	0.643 (0.512–0.755)	0.706	0.691
Classifier only	A	31/11/6/18	0.838 (0.689–0.923)	0.738 (0.589–0.847)	0.785	0.742
Classifier only	B	36/6/10/14	0.783 (0.644–0.877)	0.857 (0.722–0.933)	0.818	0.758

**Table 6:** Study-level CVS counts on the eight-study feasibility set. Values are exploratory research indicators, not diagnoses. Study identifiers are anonymized. “GT+/GT−/GT?” are reference CVS-positive/negative/unsure lesion counts; Pathway columns give CVS-positive over total detected (percentage). “Clinical MS” is the label from the full clinical workup. Both pathways report more CVS-positive clusters than the reference (80 and 85 versus 56), reflecting unmatched detections and recall-oriented operating points; the study-level indicator values should be read together with this overcounting and with the fact that the detector and thresholds rule were configured on these same eight studies. See Section 4.4 and Section 5.

Study	GT+	GT−	GT?	Pathway A +	Pathway B +	Clinical MS
1	9	4	2	16/26 (62%)	14/26 (54%)	MS+
2	9	2	1	7/11 (64%)	8/11 (73%)	MS+
3	15	2	0	22/24 (92%)	21/24 (87%)	MS+
4	18	2	3	24/28 (86%)	24/28 (86%)	MS+
5	1	10	5	7/21 (33%)	11/21 (52%)	MS−
6	0	4	1	1/7 (14%)	1/7 (14%)	MS−
7	4	4	1	1/5 (20%)	2/5 (40%)	MS−
8	0	13	1	2/4 (50%)	4/4 (100%)	MS−
Total	56	41	14	80	85	—

## Data Availability

The clinical brain MRI data analyzed in this study are not publicly available owing to patient-privacy, ethical, and institutional restrictions governing the further use of health-related personal data (EKNZ Project-ID 2025-02200), and cannot be redistributed. The source code developed for this study is not publicly available. The preprocessing, detection, clustering, and CVS-assessment configurations are described in detail in the Materials and Methods and the Appendices to support methodological reproducibility.

## Abbreviations

The following abbreviations are used in this manuscript:

MS	Multiple sclerosis
CVS	Central vein sign
SWIp	Susceptibility-weighted imaging with phase enhancement
FLAIR	Fluid-attenuated inversion recovery
MRI	Magnetic resonance imaging
DICOM	Digital Imaging and Communications in Medicine
UID	Unique identifier
IoU	Intersection over union
NMS	Non-maximum suppression
mAP	Mean average precision
TE	Echo time
TR	Repetition time
EKNZ	Ethikkommission Nordwest- und Zentralschweiz
HFG	Swiss Human Research Act
HFV	Swiss Human Research Ordinance
LUKS	Luzerner Kantonsspital (Lucerne Cantonal Hospital)

## Appendix A. Anatomical Tiling Parameters

The full-slice dataset was transformed into a tiled dataset in five steps while keeping the same studies in the same train/validation splits. (1) *Brain mask*: Otsu thresholding separated brain from background; the mask was cleaned by morphological closing (5 × 5 kernel), retention of the largest connected component, and morphological opening (3 × 3 kernel); a tight bounding box was computed and slices with a brain box under 100 pixels in either dimension were skipped. (2) *Tile placement*: the brain box was split into a 2 × 2 grid with 25% overlap; tiles with less than 30% brain coverage were discarded (most axial slices produced four valid tiles). (3) *Label remapping*: each label was intersected with each tile and assigned only if at least 50% of its area fell inside, then clipped and renormalized to tile coordinates. (4) *Dataset generation*: tiles were written as grayscale PNGs preserving bit depth, with traceable names. (5) *Split generation*: the lesion-positive validation subset contained only lesion-bearing tiles; the balanced training subset kept all positive tiles and sampled negatives (negatives within ±2 slices of any lesion excluded; negatives capped at twice the positives per study).

## Appendix B. Pathway A Implementation

Detector boxes were grouped into lesion clusters (bounding-box IoU ≥ 0.1 and slice gap ≤ 5), the highest-confidence slice per cluster was selected, its box expanded around the lesion centre (padding factor 2.0), squared, and resized to 320 × 320. The two-class training set was balanced by oversampling (CVS-negative 948; CVS-positive 551 → 948). Augmentation was mild (rotation ±5°, horizontal flip p=0.5, HSV value 0.1). The final classifier was a fine-tuned YOLOv26m classification model (320 × 320 input, 100 epochs, batch size 32, fixed seed). The deployed decision threshold is 0.35, selected from a sweep over 596 validation crops.

**Table A1:** Pathway A three-class classifier top-1 accuracy. The three-class setup performed only modestly and the weighted loss reduced accuracy further.

Three-Class Model	Top-1 Accuracy
YOLOv11m-cls	0.621
YOLOv26m-cls	0.581
YOLOv11m-cls, weighted CE	0.552

**Table A2:** Pathway A two-class classifier top-1 accuracy. Removing the no_lesion class improved performance; the balanced YOLOv26m-cls model was selected.

Setup	Model	Top-1 Accuracy
Unbalanced	YOLOv26m-cls	0.747
Unbalanced	YOLOv11m-cls	0.743
Balanced (oversampled)	YOLOv26m-cls	0.753

**Table A3:** Pathway A threshold sweep over 596 validation crops (CVS-positive recall, precision, CVS-negative recall). The deployed threshold of 0.35 favours CVS-positive recall.

Threshold	CVS+ Recall	Precision	CVS− Recall
0.20	0.939	0.561	0.416
0.25	0.902	0.604	0.530
0.30	0.856	0.646	0.627
0.35	0.833	0.683	0.693
0.40	0.795	0.702	0.732
0.50	0.652	0.758	0.834
0.60	0.511	0.808	0.904

## Appendix C. Pathway B Implementation

The vein-presence classifier is a YOLO classification model trained on the same 320 × 320 crops; its target is the has-vein attribute from the four-reader consensus (strict majority; ties treated as unsure). The segmentation model is a U-Net with a ResNet-34 encoder taking a single-channel 320 × 320 crop and producing a per-pixel vein probability; pixels above 0.5 are labelled vein and only the largest connected component is kept. Segmentation targets come from a single selected reader annotation (the most complete: bounding box, lesion outline, and vein polyline), rasterized to a binary mask at 15-pixel thickness, with categorical attributes overwritten by the consensus vote. Centrality is computed per Equation (1).

## Appendix D. Full-Slice Detection Results and End-to-End Configuration

**Table A4:** Paired full-slice versus tiled (merge-back) detection for each architecture. Tiling increases recall for three of four detectors and improves mAP@0.50 markedly for YOLOv12m and RT-DETR, but does not raise mAP@0.50 for YOLOv11m or YOLOv26m: the benefit is architecture-dependent. The deployed pipeline uses the tiled YOLOv12m detector.

	mAP@0.50			Recall	
Architecture	Full-Slice	Tiled		Full-Slice	Tiled
YOLOv11m	0.485	0.466		0.471	0.583
YOLOv12m (deployed)	0.298	0.482		0.364	0.643
YOLOv26m	0.457	0.248		0.465	0.414
RT-DETR	0.252	0.432		0.350	0.806

**Table A5:** End-to-end pipeline configuration used for the eight-study feasibility evaluation.

Setting	Value
Detector	Tiled YOLOv12m
Image size	768
Detection confidence threshold	0.10
Detection IoU threshold	0.50
Tile grid/overlap	2 × 2/25%
Cross-tile NMS IoU	0.30
Cluster neighbour gap	5 slices
Cluster IoU/centre distance	0.10/32 pixels
Minimum boxes per cluster	2
Crop size/padding factor	320 × 320/2.0
Pathway A CVS (classifier) threshold	0.35
Pathway B vein-presence probability threshold	0.35
Pathway B segmentation mask threshold	0.50
Pathway B segmentation post-processing	Largest connected component
Pathway B centrality decision threshold	0.733
Centrality-threshold selection	Youden index on clean reader-traced geometry from 519 lesions

Pathway B uses three separate thresholds at different processing stages. A probability threshold of 0.35 is applied to the vein-presence classifier as a high-recall gate, a threshold of 0.50 binarizes the U-Net vein-probability map, and a threshold of 0.733 is applied to the resulting centrality score. These thresholds operate on different model outputs and are therefore not directly comparable.

**Table A6:** Sensitivity of the end-to-end run to the detector confidence threshold. All other pipeline parameters were held fixed at the deployed configuration (Appendix D Table A5). “Reference lesions matched” counts distinct reference lesions and defines the detection rate; “unmatched clusters” counts predicted clusters matching no reference lesion. The two columns do not sum to the cluster total because two reference lesions were each matched by two predicted clusters at the deployed operating point. “Ind. A/B” is the number of the eight studies flagged MS-indicator-positive by each pathway. Thresholds above 0.40 are omitted: no cluster retained any box at those confidences.

Conf.	Clusters	Ref. Matched	Unmatched	Det. Rate	A +	B +	Ind. A/B
0.10 (deployed)	126	78	46	70.3%	80	85	4/5
0.15	119	78	39	70.3%	74	79	4/5
0.20	88	62	25	55.9%	57	60	4/4
0.25	55	42	12	37.8%	41	41	2/2
0.30	28	24	3	21.6%	24	26	2/2
0.35	10	10	0	9.0%	10	10	1/1
0.40	4	4	0	3.6%	4	4	0/0
Reference	—	111	—	—	56	56	—
